# Partizipation gestalten – digitale Gesundheitskompetenz fördern: Erfahrungen aus der Entwicklung eines Kursprogramms für Menschen mit chronischen Erkrankungen

**DOI:** 10.1007/s00103-025-04015-7

**Published:** 2025-02-07

**Authors:** Gabriele Seidel, Anabel Bornemann, Antje Meyer, Jürgen Kretschmer, Jens Krug, Marie-Luise Dierks

**Affiliations:** 1 Institut für Epidemiologie, Sozialmedizin und Gesundheitssystemforschung, Medizinische Hochschule Hannover, Carl-Neuberg-Straße 1, 30625 Hannover, Deutschland; 2 Bundesarbeitsgemeinschaft der PatientInnenstellen, München, Deutschland; 3 BARMER, Wuppertal, Deutschland

**Keywords:** Digitalisierung im Gesundheitswesen, Patientenbeteiligung, Nutzerorientierung, Patientenschulung, Projektmanagement, Digitalisation in the healthcare system, Patient participation, User orientation, Patient education, Projectmanagement

## Abstract

**Hintergrund:**

Von Patientenorganisationen wird zunehmend ein partizipatives Vorgehen in der Gesundheitsforschung gefordert. Im Projekt „KundiG – Klug und digital durch das Gesundheitswesen“ wurde bei der Entwicklung eines Schulungsprogramms für digitale Gesundheitskompetenz (dGK) die Perspektive chronisch kranker Menschen einbezogen. Ziel dieser Arbeit ist es, aufzuzeigen, welcher personelle Aufwand mit der partizipativen Entwicklung eines Schulungsprogramms verbunden ist und welche Voraussetzungen dafür notwendig sind.

**Methoden:**

Im Projektzeitraum 04/2021–03/2023 wurde von den Forschenden ein Basiskonzept erarbeitet und in einem strukturierten Partizipationsprozess in Bezug auf Inhalte, Thementiefe, Ablauf, Didaktik und Kursmaterialien kontinuierlich weiterentwickelt. Dazu wurde ein Partizipationsmodell erstellt. Eine formative Evaluation fand im Rahmen von 8 Pilotkursen statt.

**Ergebnisse:**

Insgesamt haben 20 Personen in den Arbeitsgruppen (AGs) und bei der Evaluation mitgearbeitet, davon 6 aus der organisierten Selbsthilfe, 3 von der BARMER und 11 von der Medizinischen Hochschule Hannover. Die partizipative Zusammenarbeit war arbeitsintensiv und zeitlich eng getaktet, sodass nicht alle Mitglieder in gleicher Intensität teilnehmen konnten. Es entstand ein internetbasiertes Schulungsprogramm, das von den Teilnehmenden der Pilotkurse positive Rückmeldungen und Verbesserungsvorschläge erhielt. Im Ergebnis entstanden ein 15-stündiger Online-Kurs zur Förderung der dGK, ein 300-seitiges Kursmanual und 6 digitale barrierefreie Begleithefte.

**Diskussion:**

Partizipation bedeutete die aktive Mitarbeit aller Beteiligten im gesamten Prozess, eine hohe Termindichte, die Berücksichtigung unterschiedlicher Perspektiven und ein ständiges Bemühen um Konsens. Eine vorherige Schulung der Mitwirkenden könnte bei künftigen Projekten hilfreich sein. In Vorbereitung sind die Ausrollung des Programms und die kontinuierliche Evaluation im Kontext der Selbsthilfe.

## Abstract (en)

**Background:**

Patient organisations are increasingly calling for a participatory approach to health research. In the “KundiG – Klug und digital durch das Gesundheitswesen” project, the perspective of chronically ill people was included in the development of a training programme for digital health literacy (dGK). The aim of this work is to show the personal effort involved in the participatory development of a training programme and the prerequisites for this.

**Methods:**

During the project period (April 2021 to March 2023), a basic concept was developed by the researchers and continuously refined in a structured participatory process with regard to content, depth of topics, procedure, didactics and course materials. A participation model was created for this purpose. A formative evaluation took place as part of eight pilot courses.

**Results:**

A total of 20 people were involved in the working groups and the evaluation, including 6 from organised self-help, 3 from BARMER and 11 from the Hannover Medical School. The participatory collaboration was labour intensive and tightly scheduled, meaning that not all members were able to participate to the same extent. The result was an internet-based training programme that received positive feedback and suggestions for improvement from the participants in the pilot courses. The result was a 15-hour online course to promote dGK, a 300-page course manual and six digital, accessible booklets.

**Discussion:**

Participation meant the active involvement of all participants in the entire process, a high number of appointments, the consideration of different perspectives and a constant endeavour to reach a consensus. Prior training of the participants could be helpful in future projects. The roll-out of the programme and continuous evaluation in the context of self-help are in preparation.

## Hintergrund

Die elektronische Patientenakte (ePA), aber auch andere digitale Anwendungen, wie z. B. digitale Gesundheitsanwendungen (DiGA), Videosprechstunden, Gesundheitsplattformen und Wearables stellen viele Menschen durchaus vor Herausforderungen. Sie müssen über spezifische Fähigkeiten oder schlicht die Geräte verfügen, die für einen informierten Umgang mit den digitalen Anwendungen oder für die digitale Kommunikation mit medizinischem Fachpersonal erforderlich sind. Notwendige Fähigkeiten sind das Finden, das Verstehen, das Beurteilen und die Nutzung von digitalen Informationen, die zusammengefasst als „digitale Gesundheitskompetenz“ (dGK) bezeichnet werden [1, 2].

Die dGK allerdings ist, Bevölkerungsstudien zufolge, bei 66 % der Befragten nur unzureichend ausgeprägt [3–5], bei Menschen mit chronischen Erkrankungen liegt dieser Anteil sogar bei 77,1 %. Dies ist sehr bedeutsam, weil dGK und auch generell die GK ein erfolgreiches Krankheitsmanagement und gesundheitsbezogenes Verhalten unterstützen [6]. Am schwierigsten ist es für die Bevölkerung und insbesondere für Menschen mit chronischen Erkrankungen, die Vertrauenswürdigkeit von Informationen einzuschätzen [3]. Auch aus diesem Grund hat der Gesetzgeber bereits 2023 die Krankenkassen in Deutschland verpflichtet, für ihre Versicherten Maßnahmen zur Förderung der dGK anzubieten (§ 20k SGB V; [7]).

In diesem Kontext wurde der Grundstein für das Projekt „KundiG – Klug und digital durch das Gesundheitswesen“ mit und für chronisch kranke Menschen gelegt. In den Prozess der Projektentwicklung wurden von Beginn an Vertreter:innen der organisierten Selbsthilfe eingebunden, weil sie aufgrund ihrer Expertise als Selbstbetroffene, Ehrenamtliche oder professionelle Unterstützende die Bedürfnisse und Perspektiven chronisch erkrankter Menschen authentisch einbringen können. Diese Integration folgt dem von Patientenorganisationen geforderten [7] und inzwischen auch in der Gesundheitsforschung weitgehend anerkannten Ansatz einer aktiven Beteiligung von Betroffenen. Die Partizipation soll dazu beitragen, die Forschungsqualität zu verbessern bzw. die Forschung praxisnäher und realistischer zu gestalten. Zudem kann so das Vertrauen in die Forschungsergebnisse erhöht und ein guter Transfer in die Praxis realisiert werden [8, 9]. Da ein Fernziel der beteiligten Krankenkasse darin lag, entsprechende Angebote auch für Versicherte generell nutzbar zu machen, wurden dort tätige Mitarbeiter:innen als Vertreter:innen von Versicherten ebenfalls aktiv in den Entwicklungsprozess einbezogen.

Zu den Projektpartnern gehörten die Bundesarbeitsgemeinschaft Selbsthilfe e. V. (BAG SELBSTHILFE), die als Dachverband 121 Selbsthilfeverbände, 13 Landesarbeitsgemeinschaften und 7 Fachverbände vertritt, die Nationale Kontakt- und Informationsstelle zur Anregung und Unterstützung von Selbsthilfegruppen (NAKOS), die Selbsthilfekoordination Bayern e. V. (SeKo Bayern), die ca. 36 Selbsthilfekontaktstellen in Bayern koordiniert, die BARMER Krankenkasse und die Medizinische Hochschule Hannover (MHH).

Ziel des KundiG-Projekts war die partizipative Entwicklung eines internetbasierten, modularen Schulungsprogramms zur Förderung der dGK. Wir gehen in diesem Artikel der Frage nach, welcher personelle Aufwand mit der partizipativen Entwicklung eines Schulungsprogramms verbunden ist und welche Voraussetzungen dafür notwendig sind, wie sich der partizipative Prozess gestaltet. Wir beschreiben zudem die Ergebnisse und Erfahrungen und diskutieren die Implikationen. Die Ergebnisse der standardisierten und anonymisierten Evaluation der Teilnehmenden sind in einem anderen Artikel veröffentlicht [10].

## Methoden

Das Basiskonzept für das Schulungsprogramm wurde von Forschenden erarbeitet und in einem strukturierten Partizipationsprozess, der auch die Evaluation von Pilotkursen umfasste, mit allen Beteiligten weiterentwickelt. Im Folgenden werden die partizipative Umsetzung des Projektes und speziell die Partizipation bei der Evaluation beschrieben.

### Partizipative Umsetzung des Projektes

Die Projektbeteiligten haben ein partizipatives Vorgehen umgesetzt, indem die Fach- und Lebensperspektiven der Betroffenen systematisch erfasst und einbezogen wurden. Dabei waren die Vorarbeiten von PartNet, dem Netzwerk für Partizipative Gesundheitsforschung, grundlegend, in dem Strategien und Konzepte zur partnerschaftlichen Zusammenarbeit zwischen Wissenschaftler:innen, Praktiker:innen, der Zivilgesellschaft und Expert:innen aus eigener Erfahrung bearbeitet werden [11]. Entsprechend sollte im KundiG-Projekt die Relevanz der Themenfelder der dGK erfasst, die Verständlichkeit der Inhalte gefördert und die Akzeptanz des Kursprogramms insgesamt gestärkt werden.

Der Partizipationsprozess selbst orientierte sich zunächst an den vorliegenden theoretischen Modellen, die in unterschiedlicher Differenzierung, aufeinander bezogene, in der Regel aufeinander aufbauende Stufen der Partizipation beschreiben, von den Vorstufen (Informieren, Meinung erfragen und beraten lassen) bis hin zur Mitbestimmung, der partiellen oder schließlich vollständigen kooperativen Entscheidung im Forschungsprozess [12].

Bei der Entwicklung des Schulungsprogramms zeigte sich, dass in den verschiedenen Projektphasen die Ebenen der Partizipation themenbezogen unterschiedlich zum Tragen kommen und durchaus im Sinne eines Kreislaufs immer wieder durchlaufen werden.

**Abb. 1:**
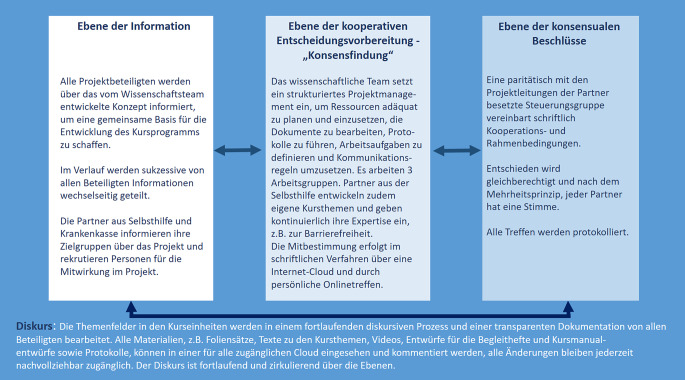
Partizipationsmodell im „KundiG-Projekt“ (eigene Darstellung)

Für die folgende Darstellung der Partizipation im Projekt haben wir deshalb den „Diskurs“ explizit in unser Partizipationsmodell (Abb. 1) aufgenommen und rekurrieren zudem auf die Ebenen „Information“, „kooperative Entscheidungsvorbereitung – ’Konsensfindung’“ sowie „konsensuale Beschlüsse“:

#### Diskurs. 

Im Prozess wurde ein regelmäßiger, auf Augenhöhe zwischen allen Beteiligten stattfindender Diskurs auf unterschiedlichen Ebenen etabliert, dafür wurden Strukturen und Methoden eingesetzt, die alle Arbeitsphasen durchzogen.

#### Ebene der Information.

Das Wissenschaftsteam informierte die potenziellen Selbsthilfepartner und eine potenzielle Krankenkasse über ein mögliches Konzept für die Unterstützung der dGK chronisch erkrankter Menschen. Dieses Konzept basierte auf einer Literaturrecherche in einschlägigen Datenbanken [13–15]. Bei Projektstart informierten auch die einbezogenen Selbsthilfepartner ihre aktiven Mitglieder, ebenso die beteiligte Krankenkasse. Die Partner rekrutierten zudem weitere Personen in ihrem Umfeld, die sich aktiv an der Gestaltung der Kurse und an der Evaluation beteiligen würden.

#### Ebene der kooperativen Entscheidungsvorbereitung – „Konsensfindung“.

Die weitere Konzeption, die Entwicklung aller Inhalte und die Evaluation erfolgten im Prozess in 3 Arbeitsgruppen: (a) zur Konsentierung der Kursinhalte und die dazu erforderliche Beobachtung und Reflexion der Kursdurchführung, (b) zur Entwicklung eines Kursmanuals und (c) zur Entwicklung der Kursbegleithefte.

Die Mitbestimmung wurde in den paritätisch besetzten Arbeitsgruppen umgesetzt, zunächst im schriftlichen Verfahren im Sinne der Kommentierung von Vorlagen (Dokumente, Folien, Videos) über eine Internet-Cloud. Das wissenschaftliche Team führte alle Kommentare und Änderungsvorschläge zu den Dokumenten in einer Tabelle zusammen, integrierte diese und führte die überarbeiteten Versionen zurück in den Bearbeitungsprozess in der Cloud. Waren Änderungsvorschläge strittig, wurden diese in einem Online-Treffen mit den Vertreter:innen der Projektpartner mit Blick auf Konsensfindung diskutiert. In allen AGs wurde nach dem Mehrheitsprinzip entschieden, alle Beteiligten hatten eine Stimme.

#### Ebene der konsensualen Beschlüsse.

Die Projektpartner steuerten und entschieden über alle Schritte und Produkte im Projekt in einer von ihnen etablierten Steuerungsgruppe, in der jeweils die Projektleitungen aller Partner vertreten waren. Sie entschieden gleichberechtigt und nach dem Mehrheitsprinzip mit jeweils einer Stimme, die Treffen erfolgten in Online-Sitzungen und wurden protokolliert.

### Formative Evaluation und Partizipation

In dem skizzierten partizipativen Vorgehen wurden die Kurseinheiten und Materialien entwickelt und im Prozess fortlaufend einer formativen Evaluation unterzogen. Zur Überprüfung des entwickelten Kursprogramms dienten 8 Pilotkurse (48 Termine), die von 2 in der Moderation und Lehre erfahrenen Wissenschaftler:innen der MHH geleitet und durchgeführt wurden.

Die Rekrutierung der Kursteilnehmenden, die explizit alle aus den Selbsthilfeorganisationen bzw. -gruppen kommen sollten, war Aufgabe der 3 Institutionen der Selbsthilfe. Sie luden alle Interessierten ein und entschieden in eigener Verantwortung darüber, wer an den Kursen teilnehmen konnte. Das Teilnahmemanagement war Aufgabe der MHH, es erfolgte telefonisch und per E‑Mail.

Die formative Evaluation bestand aus den 3 Elementen „systematische Beobachtungen“, „Reflexionsgespräche“ und „qualitative Kurzbefragungen von Kursteilnehmenden“ und wurde in 2 Zyklen umgesetzt: Im ersten Zyklus fanden 3 Kurse mit jeweils 6 Kurseinheiten statt. Diese waren so gestaffelt, dass alle 3 Kurse in einer Woche an je unterschiedlichen Wochentagen begannen und so jede Einheit 3‑mal pro Woche mit je unterschiedlichen Teilnehmenden durchgeführt werden konnte. Erste Anpassungen der Materialien, der Kursinhalte, der zeitlichen Struktur und der Didaktik erfolgten unmittelbar nach jeder Einheit. So konnten erste Überarbeitungen in den folgenden Kurseinheiten und Gruppen nicht nur umgesetzt, sondern auch direkt überprüft und die Wirkung der Änderungen nachvollzogen werden.

Im zweiten Zyklus (weitere 5 Kurse mit ebenfalls 6 Kurseinheiten) wurden alle Elemente des Kurses weiter überprüft und ggf. aktualisiert. Die AG arbeitete daran, das Kurskonzept so zu manualisieren, dass interessierte weitere Mitglieder der Selbsthilfe auf Grundlage des Kursmanuals und entsprechender Schulung der Kursleitungen die KundiG-Kurse eigenständig durchführen können. Ein weiterer Schwerpunkt lag in diesem Zyklus auf der Entwicklung und Erprobung eines Begleitheftes für jede Kurseinheit, das die Teilnehmenden im Selbststudium unterstützt und ihnen die Möglichkeit gibt, die Inhalte während des Kurses und auch darüber hinaus zu vertiefen.

#### Teilnehmende Beobachtungen.

**Abb. 2:**
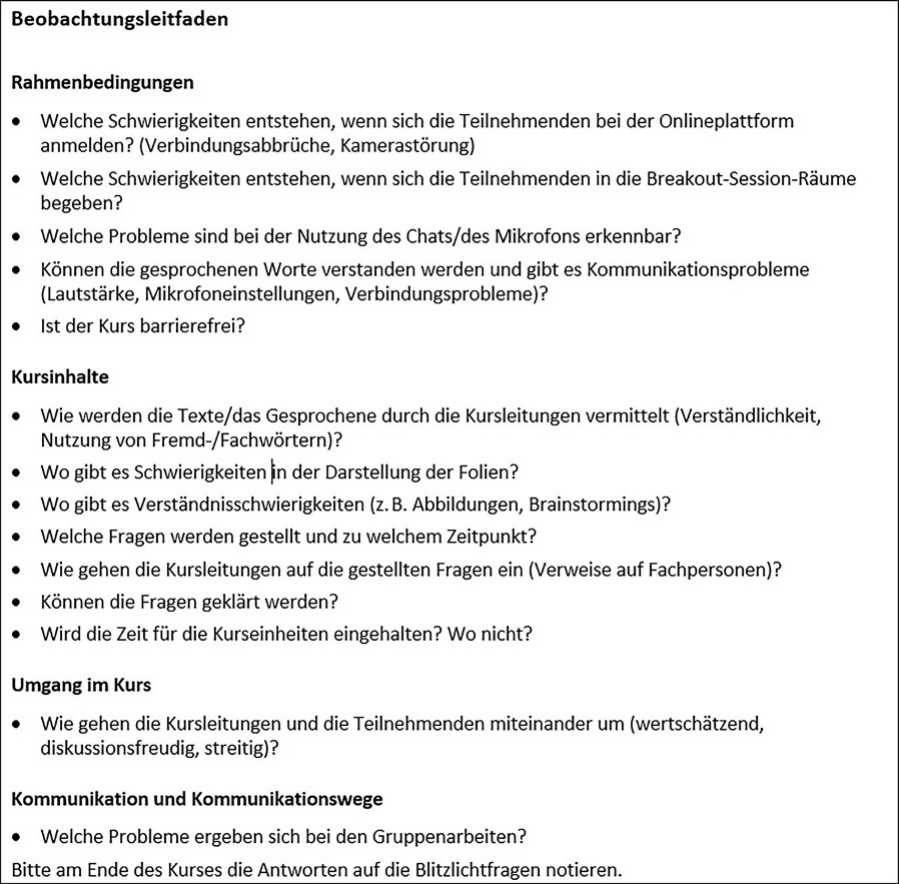
Beobachtungsleitfaden (eigene Darstellung)

Eine teilnehmende Beobachtung erfolgte an jedem der 48 Kurstermine durch je 2–3 Personen aus der „AG für die Konsentierung der Inhalte“ (jeweils Personen aus der Selbsthilfe, der Wissenschaft und der BARMER), ein gemeinsam entwickelter Beobachtungsleitfaden (Abb. 2) diente der Dokumentation. Die Beobachtenden waren in den digitalen Kursen zugeschaltet, die Kursteilnehmenden waren informiert und hatten ihr Einverständnis erklärt, im Prozess waren die Kameras der Beobachtenden deaktiviert. Für die Analyse wurden die Hauptbereiche des Beobachtungsleitfadens als Kategorien genutzt. Die erhobenen Daten wurden softwaregestützt inhaltlichen, überwiegend deduktiven Kategorien zugeordnet und im Sinne der qualitativen Inhaltsanalyse nach Mayring ausgewertet (MAXQDA, Version 2020, VERBI Software GmbH, Berlin, Deutschland; [16]).

#### Strukturierte moderierte Reflexionsgespräche.

**Abb. 3:**
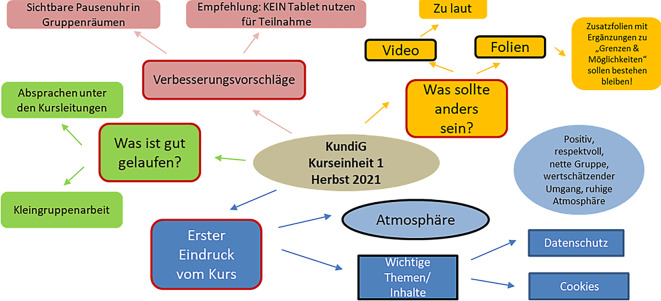
Muster eines „Knowledge-Mappings“ als Ergebnis des moderieren Reflexionsgesprächs (eigene Darstellung)

Die strukturierten Reflexionsgespräche fanden unmittelbar nach jedem Kurstermin statt, sie dienten dem Austausch der Erfahrungen der Kursleitungen und der Beobachtungspersonen und insbesondere einer vertieften Auseinandersetzung mit Inhalten und Didaktik. Beteiligt waren hier 1 Moderatorin aus der Wissenschaft, die beiden Kursleitungen, 1 Beobachterin aus der Wissenschaft und in der Regel 2 Beobachter:innen der weiteren Projektbeteiligten. Das Gespräch wurde auf Basis eines Leitfadens durchgeführt, im Audiomitschnitt aufgezeichnet und anschließend im Wortlaut regelgetreu transkribiert [17]. Der erste Analyseschritt erfolgte über ein „Knowledge-Mapping“ ([18]; beispielhaft dargestellt in Abb. 3), über das Änderungsvorschläge und Anmerkungen für eine systematische Aufarbeitung transparent dargestellt wurden, es orientierte sich an den Fragen aus dem Leitfaden.

#### Qualitative Rückmeldungen der Kursteilnehmenden.

Zur Erfassung der Eindrücke der Kursteilnehmenden bezüglich der inhaltlichen und didaktischen Umsetzung der Themenfelder kam in einem letzten Schritt eine „Blitzlichtrunde“ am Ende des letzten Kurstermins zum Einsatz. Gefragt wurde: „Wie war der Kurs für Euch?“ „Was hat Euch besonders gut gefallen?“ Und: „Habt Ihr Tipps für eine Veränderung des Kurses?“ Diese Rückmelderunde wurde durch die Kursleitungen moderiert, die Aussagen der Teilnehmenden von den Beobachtenden dokumentiert und paraphrasierend in das Beobachtungsprotokoll übernommen.

## Ergebnisse

Im Folgenden werden Erfahrungen mit dem partizipativen Prozess und Ergebnisse des KundiG-Projekts dargestellt.

### Die partizipative Zusammenarbeit bei der Entwicklung des Kursprogramms

An dem zeitlichen Umfang der Projektentwicklung und den eingesetzten personellen Ressourcen lässt sich zeigen, dass die partizipative Zusammenarbeit für alle Beteiligten arbeitsintensiv ist. So traf sich die *Steuerungsgruppe* im Projektzeitraum von April 2021–März 2023 online 13-mal, die AGs trafen sich 3‑ bis 6‑mal, wobei zu berücksichtigen ist, dass alle Treffen vor- und nachbereitet werden müssen. 20 Personen haben dabei in unterschiedlicher Intensität aktiv in AGs und bei der Evaluation mitgearbeitet, davon 6 Personen von den 3 Selbsthilfepartnern, 3 von der BARMER und 11 von der MHH.

Die 3 AGs bestanden über den gesamten Projektzeitraum hinweg aus 5–10 Personen. Grundsätzlich waren bei allen Aktivitäten alle Projektpartner beteiligt, durchaus in unterschiedlicher Intensität. Lediglich die Wissenschaftler:innen waren konstant in den jeweiligen AGs vertreten.

Die Mitglieder der *AG für die Konsentierung der Inhalte* trafen sich 6‑mal zur Erarbeitung der Themenfelder, waren auch aktiv an der Evaluation der 8 Kurse in allen 48 Kurseinheiten als Beobachtende beteiligt, ebenso an den 48 Reflexionsgesprächen (Selbsthilfepartner: 45 Beobachtungen, 27 Reflexionsgespräche; BARMER: 3 Beobachtungen, 3 Reflexionsgespräche; MHH: 39 Beobachtungen, 48 Reflexionsgespräche).

Die *AG für die Erstellung des Kursmaterials*, wie z. B. der PowerPoint-Folien und der Texte, traf sich online 6‑mal und arbeitete zudem über 9 Monate hinweg regelmäßig über die Cloud zusammen. Auch die *AG für die Erstellung der 6 Begleithefte* arbeitete kontinuierlich 9 Monate über die Cloud zusammen und traf sich online 3‑mal im Projektzeitraum.

Der geleistete Arbeitsaufwand lässt sich auch anhand der Kommentare und Änderungsvorschläge zu den Materialien illustrieren. So haben die Mitwirkenden aus der Selbsthilfe 537 Textstellen des Materials kommentiert und Verbesserungsvorschläge integriert (Kursmanual 297 Textstellen; Foliensätze 98 Textstellen; Begleithefte 142 Textstellen). Im weiteren Konsensprozess wurden zwischen 77 % und 88 % der Vorschläge übernommen, im Durchschnitt 84 %, nur 16,4 % aller Textstellen wurden begründet abgelehnt. In diesem Prozess entwickelte sich beispielsweise auch ein genuin nicht im Konzeptentwurf enthaltener Themenschwerpunkt „Digitale Selbsthilfe“, ebenso Hinweise auf Apps und Videomaterial aus der Selbsthilfe.

**Abb. 4:**
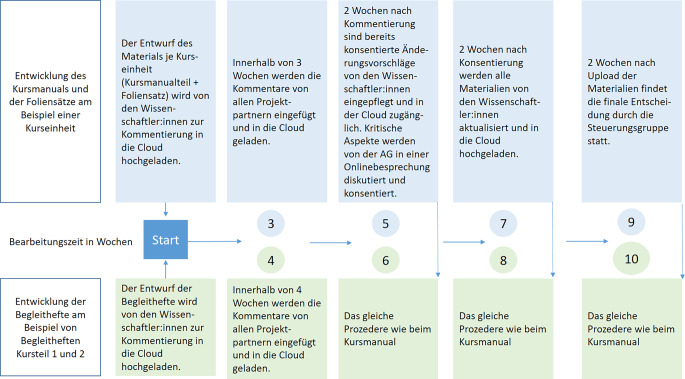
Bearbeitung der Kursmaterialien im zeitlichen Verlauf (eigene Darstellung)

Das zeitliche Vorgehen wird in Abb. 4 am Beispiel der Entwicklung des Kursmanuals und der Begleithefte für eine Kurseinheit illustriert, dieses Prozedere wiederholte sich im Projekt 6‑mal für das Kursmanual und 3‑mal für die Begleithefte.

Dieses zeitlich eng getaktete Prozedere war notwendig, um das Projektziel in der vereinbarten Zeit zu erreichen, führte jedoch auch dazu, dass nicht immer alle AG-Mitglieder in der gleichen Intensität Rückmeldungen geben konnten oder an allen AG-Treffen beteiligt waren. In der Regel waren die Wissenschaftler:innen, die mit der jeweiligen Kurseinheit beschäftigt waren, bei allen Besprechungen anwesend. Das bedeutet: Im Ergebnis waren die AGs paritätisch im Sinne von „alle Projektpartner waren vertreten“ besetzt, aber nicht paritätisch im Sinne der Personenanzahl. Dies wurde mithilfe des transparenten Vorgehens aufgefangen und vor allem durch die diskursive Ausrichtung aller Prozesse, die nicht zuletzt zu einer offenen Arbeitsatmosphäre führten.

### Die Lehr- und Lernmethoden

Für die Pilotkurse wurde eine Didaktik gewählt, die neben reinem Wissenserwerb auf praktisch erprobbare Elemente und den kontinuierlichen Austausch mit Leitungspersonen und anderen Teilnehmenden setzt [14, 19, 20]. Deshalb gab es in abwechslungsreicher Zusammenstellung Kurzvorträge, unterstützt durch PowerPoint-Folien, Rückmelderunden, Videos [21], Podcasts [22], Fallbeispiele [23], Brainstormings [24], Aufrufe, Demonstrationen, Übungen in Gruppen- und Partnerarbeit in Breakout-Rooms (Aufteilung einer Gruppe in kleinere Gruppenräume bei Zoom; [25, 26]), Quizfragen [27], Aufgaben zum Selbststudium [28] und schriftliche Informationen.

Aus Sicht der Beobachter:innen der Kurse hat sich dieser Methodenmix bewährt. Aus ihrer Perspektive waren insbesondere der Wechsel zwischen aktiven und passiven Anteilen und die thematischen Einführungen über Folienpräsentationen für den Lernprozess förderlich. Ergänzend haben sie angemerkt, dass es durchaus wichtig ist, relevante Inhalte mehrfach zu präsentieren und damit zu wiederholen, um so das Verständnis bei den Teilnehmenden zu fördern.

### Barrierefreiheit

Wichtige Anpassungen erfolgten vor allem auf Anregung der 3 Selbsthilfepartner in Bezug auf die Barrierefreiheit. Sowohl in den Beobachtungsprotokollen wie auch in den Reflexionsgesprächen wurden zahlreiche Anregungen formuliert. Ergänzt wurden infolge z. B. Videountertitel. Modifiziert wurden alle Foliensätze und die elektronisch verfügbaren Begleithefte mit formatierten Informationen (Quicklinks) und beschreibenden Informationen zu Links und Abbildungen (Screenreader-Funktion für Menschen mit Sehbehinderung), die Schriftgrößen wurden angepasst.

### Technische Aspekte und Umgang der Teilnehmenden mit dem Online-Format

Um das Wissen und die Fähigkeit auszuweiten, den Computer und die Videoplattform Zoom effizient zu nutzen, war bereits im Konzept eine vor Kursbeginn stattfindende „Session Zero“ konzipiert worden. Diese sollte dazu führen, die Akzeptanz der Teilnehmenden zu erhöhen, ihr Interesse an der Teilnahme zu stärken, den Anteil derer, die den Kurs abbrechen, zu reduzieren und so zur Stärkung der dGK beizutragen [29].

Die „Session Zero“ beinhaltet einen Überblick über den Kurs und die Kursregeln (z. B. Mikrofon aus, Kamera an), technische Einstellungen (z. B. Screenreader, Tastenkombinationen) und die Möglichkeit, Fragen stellen zu können, insbesondere zu technischen Aspekten, und die Möglichkeit, die Funktionen unter Anleitung auszuprobieren. Dies hat sich im Prozess als hilfreich erwiesen. Die Session Zero und die Erklärungen der Kursleitungen zum Umgang mit der automatischen Raumzuweisung (Breakout-Rooms) in Zoom waren für die Kursteilnehmenden hilfreich und in der Regel verständlich. Allerdings gab es immer wieder Situationen, in denen Personen, die an den im Konzept vorgesehenen Gruppenarbeiten in dafür vorgesehenen „Breakout-Räumen“ teilnehmen wollten, von der Technik überfordert waren. So gelangten sie manchmal nicht in die Breakout-Räume oder verließen diese vorzeitig. Einzelne Teilnehmende konnten zudem aufgrund technischer Probleme nicht an der Kleingruppenarbeit teilnehmen.

### Die Interaktion der Teilnehmenden in den Kursgruppen

Die Kommunikation im Pilotkurs fand auf verschiedenen virtuellen Wegen statt – im Chat, durch virtuelle Rückmeldungen und durch aktive mündliche Beiträge. Die Atmosphäre und der Umgang innerhalb der Gruppen wurden von den Beobachtenden und den Kursleitungen durchweg positiv wahrgenommen. Sie beschrieben diese als „aktiv“, „diskussionsfreudig“, „wertschätzend“ und „gemeinschaftlich“.

### Die Kommunikation der Kursleitungen mit den Teilnehmenden

Die Beobachtenden haben auf viele positive Verhaltensweisen der Kursleitungen hingewiesen, wie „wertschätzend“, „souverän“, „respektvoll“, „ruhig“ und „einfühlsam“. Kursinhalte wurden verständlich, langsam und ruhig vermittelt, Fotos, Illustrationen und Fremdwörter gut erläutert. Offene Fragen der Teilnehmenden konnten in der Regel sehr gut beantwortet werden. An einigen Stellen wurden Zeitprobleme beobachtet, weil es zahlreiche Fragen der Teilnehmenden gab, die in der zur Verfügung stehenden Zeit nicht immer gut beantwortet werden konnten.

Durch das Prinzip, dass jede Kurseinheit von 2 Kursleitungen gestaltet wird, wirkte der im Verlauf vorgesehene Wechsel zwischen diesen Leitungen „abwechslungsreich“ und „erfrischend“. Es gab jedoch auch kritische Anmerkungen. So funktionierte die Aufteilung der Präsentationen durch die Kursleitungen an einigen Stellen nicht reibungslos und Nachrichten im Chat wurden teilweise übersehen. In den Breakout-Rooms zur Kleingruppenarbeit wurde eine noch aktivere Moderation durch die Kursleitungen gewünscht.

### Die Bewertung des Pilotkurses in den abschließenden Blitzlichtrunden

Zur Bewertung des Kurses liegen Hinweise von 95 Teilnehmenden aus den 8 KundiG-Kursen vor. Am jeweils letzten Kurstag wurden die Erfahrungen der Kursteilnehmenden, die sich rückblickend auf den ganzen Kurs bezogen, durch eine Rückmelderunde (Blitzlicht) erhoben und von den Beobachtenden dokumentiert. Dabei wurden in Summe 164 thematisch unterschiedliche Rückmeldungen festgehalten, 76 % der Nennungen (*n* = 125) bezogen sich auf positive Aspekte, knapp 24 % (*n* = 39) auf kritische Hinweise oder Verbesserungsvorschläge.

Zu den positiven Rückmeldungen gehört zunächst der Wissenszuwachs (*n* = 35). Das neue Wissen hat nach Aussagen der Teilnehmenden ihre Hemmschwelle, sich mit digitalen Gesundheitsthemen auseinanderzusetzen, gesenkt und ihre anfängliche Skepsis abgebaut. Sie empfanden „viele gute Informationen“, „spannend und interessant“. Zudem wurden die Vielfalt der Themen und die Hinweise auf seriöse Internetseiten positiv hervorgehoben (*n* = 18) sowie der Austausch und die Diskussionen (*n* = 12). So formulierten einige Teilnehmende, dass sie alle ihre Fragen stellen konnten und diese auch beantwortet wurden. Ferner gab es Lob für die Methodik im Kurs (*n* = 8) und für die Art und Weise, wie die Kursleitungen den Kurs durchgeführt haben (*n* = 7), z. B. „verständnisvoll“, „interessiert“ und „gute Zusammenarbeit zwischen den Kursleitungen“. Auch schätzten einige Teilnehmenden es positiv ein, dass die Informationen verständlich präsentiert wurden (*n* = 5) und zum Weiterinformieren anregen sowie weitergegeben werden konnten (*n* = 6). Einige meldeten zudem zurück, dass die Organisation um den Kurs gut gelang (*n* = 9) und dass die Online-Teilnahme gut funktionierte (*n* = 3). Auch die Inhalte der Begleithefte wurden gelobt (*n* = 22), z. B.: „Die Erklärungen in den Heften und Internetlinks zu den Videos und Informationsseiten helfen sehr gut dabei, sich weiter zu informieren.“

Es wurden aber auch kritische Aspekte genannt (24 %), z. B. „die Informationen waren für die Teilnehmenden erschlagend“. So gab es kritische Rückmeldungen zur „Informationsflut“ (*n* = 14), zum Versandzeitpunkt der Begleithefte (*n* = 9) und zum Zeitumfang des Kurses (*n* = 5). Selten geäußerte kritische Rückmeldungen bezogen sich auf unklare Aufgabenstellung für die Kleingruppen, die Länge eines Quiz, den Wunsch nach mehr Praxisbeispielen und fehlende Barrierefreiheit bei Videos. Zudem wünschten sich die Teilnehmenden weniger Informationen zum Thema Telematikinfrastruktur und zur Selbsthilfestruktur und mehr Informationen zur ePA, DiGA und Apps. Diese von den Beobachter:innen festgehaltenen Aussagen spiegeln sich auch in den Ergebnissen der standardisierten anonymisierten Evaluation wider [10].

Zur Charakterisierung der Kursteilnehmenden und damit zur Einordnung der oben dargestellten Ergebnisse können soziodemografische Angaben von 77 Teilnehmenden der 95 Teilnehmenden herangezogen werden, die im Rahmen der standardisierten, anonymen Evaluation Angaben dazu gemacht haben (Tab. 1; [10]).

**Tab. 1:** Soziodemografie der Kursstartenden in den 8 KundiG-Pilotkursen; *n* = 77

Geschlecht		Weiblich	Männlich	Gesamt
		65 (84 %)	12 (16 %)	77 (100 %)
Alter	Durchschnitt	57 Jahre	67 Jahre	59 Jahre
	Median	59 Jahre	66,5 Jahre	60 Jahre
	Spannweite	59 (22–81) Jahre	29 (51–80) Jahre	59 (22–81) Jahre
	Unter 40	5 (8 %)	0	5 (6 %)
	40–49	9 (14 %)	0	9 (12 %)
	50–59	20 (31 %)	3 (25 %)	23 (30 %)
	60–69	24 (37 %)	4 (33 %)	28 (36 %)
	Über 70	7 (11 %)	5 (42 %)	12 (16 %)
Bildungsniveau	Niedrig^2^	8 (13 %)	1 (8 %)	9 (12 %)
	Mittel^3^	14 (23 %)	4 (33 %)	18 (24 %)
	Hoch^4^	40 (64 %)	7 (58 %)	47 (64 %)
Berufstätigkeit^1^	Berentet/Pension	27 (42 %)	10 (83 %)	40 (48 %)
	Teilzeit	20 (31 %)	0 (0 %)	20 (26 %)
	Vollzeit	12 (19 %)	1 (8 %)	13 (17 %)
	Hausfrau/Hausmann	9 (14 %)	1 (8 %)	10 (13 %)
	Ausbildung/Schule	1 (2 %)	0 (0 %)	1 (1 %)
	Dauerhaft krankgeschrieben	2 (3 %)	1 (8 %)	3 (4 %)
Tätigkeit im Gesundheitswesen		34 (52 %)	1 (8 %)	35 (46 %)
Alleine lebend		18 (28 %)	1 (8 %)	19 (25 %)
Selbst chronisch erkrankt		44 (68 %)	10 (83 %)	54 (70 %)
Angehörige chronisch erkrankt		20 (31 %)	1 (8 %)	21 (27 %)

^1^Mehrfachnennung möglich

^2^Volks‑/Hauptschulabschluss bzw. Polytechnische Oberschule mit Abschluss 8. oder 9. Klasse

^3^Mittlere Reife, Realschulabschluss bzw. Polytechnische Oberschule mit Abschluss 10. Klasse

^4^Fachhochschulreife/Abitur/ (Fach‑)Hochschulabschluss

### Aktueller Stand des Kurses

Am Ende des Projekts steht ein manualisierter 6‑teiliger Online-Kurs zur Förderung der dGK für jeweils 8–14 Kursteilnehmende zur Verfügung, der 2,5 h pro Woche – inklusive 2 Pausen von insgesamt 20 min, also insgesamt 15 h – dauert und von 2 Kursleitungen durchgeführt wird. Das 300 Seiten umfassende Kursmanual enthält 6 Kurseinheiten mit 22 Detailthemen, die Verankerung der Themen im Kursverlauf, die jeweiligen Lernziele, die benötigten Materialien, Hinweise zum Umgang mit dem Kursmanual, eine Checkliste für die Kursleitung mit Beispielen für die Zusammenarbeit, eine Anleitung für eine Session Zero, eine Anleitung zur Nutzung der Kommunikationsplattform Zoom, Hinweise auf Tastenkombinationen und Shortcuts für sehbehinderte Menschen und eine Kurzbeschreibung der eingesetzten Lehr- und Lernmethoden.

Für die Kursdurchführung wurden 6 PowerPoint-Präsentationen und 6 digitale Begleithefte mit insgesamt 88 Seiten unter Berücksichtigung von Aspekten der Barrierefreiheit entwickelt. Die digitalen Begleithefte enthalten Kurzinformationen, Links zu weiterführenden Informationen, Aufgaben zum Selbststudium sowie ein Glossar mit wichtigen Begriffen zu den jeweiligen Themenfeldern.

**Abb. 5:**
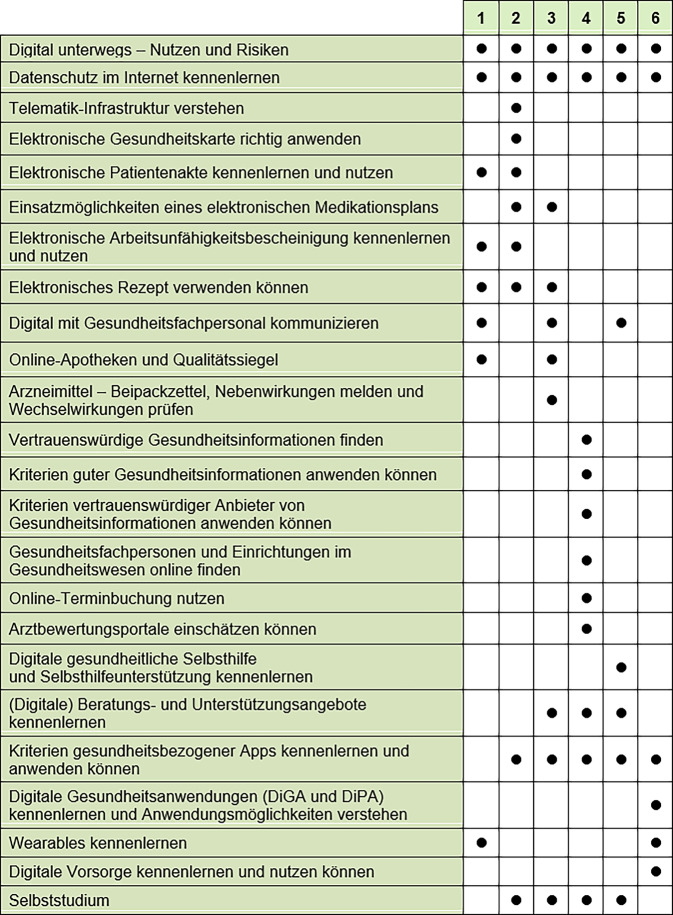
Themen des KundiG-Kurses innerhalb der 6 Kurseinheiten (Quelle: eigene Darstellung). *DiPA* digitale Pflegeanwendungen

Die Kursmaterialien sind so aufgebaut, dass z. B. auch nur einige Module angeboten werden können oder für bestimmte Zielgruppen einzelne Module besonders detailliert angeboten werden können. Abb. 5 zeigt in welchen der 6 Kurseinheiten die verschiedenen Kursthemen behandelt werden.

Die gemeinsame Arbeit wird auch nach der Pilotphase fortgesetzt. In Vorbereitung sind die Ausrollung des Programms und die kontinuierliche Evaluation im Kontext der Selbsthilfe.

## Diskussion

In der Gesundheits- und Public-Health-Forschung soll Partizipation als Mittel dienen, um die Perspektive von Bürger:innen und Patient:innen gezielt in den Forschungsprozess zu integrieren [7–9, 12]. In den Studien, an denen Bürger:innen bzw. Patient:innen bislang beteiligt waren, bewegt sich die Partizipation allerdings noch vorwiegend auf den Vorstufen der Partizipation [30]. In der praktischen Umsetzung sind viele Aspekte in Bezug auf die erforderlichen zeitlichen, finanziellen personellen und motivationalen Ressourcen zu bedenken [31, 32]. Dazu gehört für die Forschenden wie für die weiteren Beteiligten die Klärung, welche methodischen Kompetenzen sie bis zu welchem Grad in den Prozess einbringen sollten. Auch ist zu fragen, wie es gelingen kann, die Motivation der einbezogenen Bürger:innen oder Patient:innen über einen längeren Zeitraum aufrechtzuerhalten, den kontinuierlichen Dialog auf Augenhöhe zu führen, adäquate Rückmeldeschleifen einzubauen und das Spannungsverhältnis zwischen Ehrenamt und Professionalität auszubalancieren [9, 33].

Die in unserem Projekt umgesetzte Form der Partizipation war insofern erfolgreich und besonders, als von Beginn an die Selbsthilfe mit ihren Repräsentant:innen und die Vertreter:innen der Krankenkasse gemeinsam mit den Wissenschaftler:innen alle Prozesse transparent gestaltet und dokumentiert haben. Die Personen aus der Selbsthilfe waren zwar zum Teil in ihrer Rolle als Funktionäre mit Blick auf die Dissemination der Inhalte am Prozess beteiligt, sie waren aber in der Regel auch selbst von einer chronischen Erkrankung betroffen.

Die Partizipation im Forschungsprozess wurde dadurch gefördert, dass zu Beginn die Rahmenbedingungen partizipativ festgelegt, die Nutzungsrechte geklärt und die Finanzierung der beteiligten Mitwirkenden klar geregelt wurden. Dies sind einige der Kriterien, die auch Wright als Erfolgsfaktoren für Partizipation nennt [34]. Ein strukturiertes Projekt- und Zeitmanagement sowie eine Prozessmoderation in allen (Vor‑)Phasen waren für den Erfolg des Projektes unabdingbar [31]. Die einbezogenen Partner:innen konnten auch als Sprachrohre für die Gruppe der chronisch Erkrankten bzw. der Mitglieder von Selbsthilfegruppen im Prozess fungieren, in ihren Organisationen bei den Mitgliedern Interesse an dem Kurs wecken und werden auch nach der Modellphase für eine weitere Verbreitung sorgen.

Einschränkend muss allerdings betont werden, dass, wie in der Literatur berichtet, ein partizipatives Verfahren nicht immer einfach umzusetzen ist, dies wird auch aus den bislang noch wenigen Studien deutlich [9]. In unserem Projekt waren beispielsweise die zeitlichen Ressourcen der AG-Mitglieder durchaus unterschiedlich, was die Intensität der Zusammenarbeit entsprechend unterschiedlich gestaltete. Auch der Diskurs und die kooperative Entscheidungsvorbereitung „Konsensfindung“ verliefen nicht immer konfliktfrei, unterschiedliche Präferenzen, methodische Vorstellungen und strategische Interessen spielten hierbei eine Rolle.

Zudem wäre zu überdenken, was bzw. wie viel die beteiligten Mitwirkenden im Vorfeld verstehen und wissen müssen, z. B. in Bezug auf Evaluationsmethoden oder die Methodik und Didaktik der Bildungsarbeit. Dies könnte Verständnisprobleme im Verlauf auffangen, entsprechende übergreifende Schulungen könnten hier hilfreich sein [9].

## Fazit

Die unterschiedlichen Sichtweisen und Interessen führten zu einem fruchtbaren, gemeinsamen Lernprozess, der sich schließlich positiv auf die Qualität des Kursprogramms auswirkte. Ein umfassendes Qualitätssicherungskonzept ist aus Sicht der Autor:innen und der Beteiligten in der Steuerungsgruppe unabdingbar. Dazu gehört neben der Evaluation der Effekte auf die dGK der Teilnehmenden in Bezug auf Ziele, Inhalte, Didaktik und Materialien auch eine kontinuierliche Aktualisierung des manualisierten Programms unter Einbezug der Selbsthilfepartner, da sich im Verlauf der Digitalisierung des Gesundheitswesens vieles ändert bzw. zukünftig weiter ändern wird. Inwieweit die Einbeziehung von Vertreter:innen chronisch erkrankter Menschen in die Entwicklung eines Online-Kurses zur Erhöhung der dGK effektiver ist als eine nur expertengeleitete Entwicklung, kann hier allerdings abschließend nicht beantwortet werden, da eine vergleichende Evaluation nicht vorliegt.

## References

[1] Dratva J, Schaeffer D, Zeeb H (2024) Digitale Gesundheitskompetenz der Bevölkerung in Deutschland: Aktueller Stand, Konzepte und Herausforderungen. Bundesgesundheitsblatt Gesundheitsforschung Gesundheitsschutz 67:277–284. 10.1007/s00103-024-03841-5 38315221 10.1007/s00103-024-03841-5 PMC10927882

[2] Zeeb H, Pohlabeln H, Preising A, Schulz B, Naczinsky A, Kolpatzik K (2023) Schlüsselqualifikation digitale Gesundheitskompetenz – empirische Daten aus Deutschland. In: Rathmann K, Dadaczynki K, Okan O, Messer M (Hrsg) Gesundheitskompetenz, 1. Aufl. pflege – Therapie – Gesundheit. Springer, Berlin, S 377–390

[3] Schaeffer D, Berens E‑M, Gille S et al (2021) Gesundheitskompetenz der Bevölkerung in Deutschland – vor und während der Corona Pandemie: Ergebnisse des HLS-GER 2. https://pub.uni-bielefeld.de/download/2950305/2950403/HLS-GER%202_Ergebnisbericht.pdf. Zugegriffen: 24. Nov. 2024

[4] Sørensen K, Van den Broucke S, Fullam J et al (2012) Health literacy and public health: a systematic review and integration of definitions and models. BMC Public Health 12:1. 10.1186/1471-2458-12-80 22276600 10.1186/1471-2458-12-80 PMC3292515

[5] Krüger-Brand HE (2019) Digitale Gesundheitskompetenz: Datensouveränität als Ziel. Dtsch Ärztebl Int 116(10):468–473

[6] Ernstmann N, Bauer U, Berens E‑M et al (2020) DNVF Memorandum Gesundheitskompetenz (Teil 1) – Hintergrund, Relevanz, Gegenstand und Fragestellungen in der Versorgungsforschung. Gesundheitswesen 82:e77–e93. 10.1055/a-1191-3689 32698208 10.1055/a-1191-3689

[7] Arbeitsgemeinschaft im Diskursverfahren des PANDORA-Forschungsprojekts (2024) Einbeziehung von Patientenorganisationen in die digitale Gesundheitsforschung : ein Positionspapier zum gegenwärtigen Status mit Forderungen für die Zukunft. https://reposit.haw-hamburg.de/bitstream/20.500.12738/16427/3/Positionspapier_Patientenorganisationen_DigitaleGesundheitsforschung_PANDORA_2024.pdf. Zugegriffen: 12. Nov. 2024

[8] BMBF (2023) Partizipationsstrategie Forschung. https://www.bmbf.de/SharedDocs/Downloads/de/2023/partizipationsstrategie.pdf?__blob=publicationFile&v=1. Zugegriffen: 12. Nov. 2024

[9] Jilani H, Rathjen KI, Schilling I et al (2022) Handreichung zur Patient*innenbeteiligung an klinischer Forschung. Universität Bremen 10.26092/elib/1925 (Version 1.1)

[10] Seidel G, Bornemann A, Hartmann M et al (2023) Förderung der digitalen Gesundheitskompetenz durch das Kursprogramm „KundiG“. In: Welt der Krankenversicherung 4/2023. Medhochzwei,

[11] Netzwerk partizipative Gesundheitsforschung PartNet Definition – Partizipative Gesundheitsforschung. http://partnet-gesundheit.de/ueber-uns/partnet-definition/. Zugegriffen: 12. Nov. 2024

[12] Schütt A, Müller-Fries E, Weschke S (2023) Aktive Beteiligung von Patientinnen und Patienten in der Gesundheitsforschung – eine Heranführung für (klinisch) Forschende. Zenodo

[13] Seidel G, Kaiser B, Lander J, Dierks M‑L (2017) The Hannover patient university: advanced mini-med school concept and evaluation results. Health Educ J 76(1):38–51. 10.1177/0017896916647751

[14] Dierks M‑L, Lander J, Seidel G (2016) Patientenwissen vor Ort verbessern – Erfahrungen aus der Patientenuniversität an der Medizinischen Hochschule Hannover. Blickpunkte Mensch Ges Sicherh

[15] Seidel G, Dierks M‑L (2020) Die Bürger fördern. Pflege Z 73:24–27. 10.1007/s41906-020-0914-5 33100588 10.1007/s41906-020-0914-5 PMC7573522

[16] Mayring P (2015) Qualitative Inhaltsanalyse: Grundlagen und Techniken, 12. Aufl. Beltz, Weinheim

[17] Kuckartz U (2014) Qualitative Inhaltsanalyse. Methoden, Praxis, Computerunterstützung, 2. Aufl. Beltz Juventa, Weinheim

[18] Pelz C, Schmitt A, Meis M (2004) Knowledge Mapping als Methode zur Auswertung und Ergebnispräsentation von Fokusgruppen in der Markt- und Evaluationsforschung. Forum Qual Sozialforsch 5(2):35

[19] Bandura A (1977) Self-efficacy: toward a unifying theory of behavioral change. Psychol Rev 84:191–215. 10.1037/0033-295X.84.2.191 847061 10.1037//0033-295x.84.2.191

[20] Siebert H (2006) Didaktisches Handeln in der Erwachsenenbildung: Didaktik aus konstruktivistischer Sicht, 5. Aufl. ZIEL, Augsburg

[21] Findeisen S, Horn S, Seifried J (2019) Lernen durch Videos – Empirische Befunde zur Gestaltung von Erklärvideos. MedienPädagogik. 10.21240/mpaed/00/2019.10.01.X

[22] (2022) Der Selbsthilfe Podcast. https://www.bag-selbsthilfe.de/der-selbsthilfepodcast/. Zugegriffen: 12. Nov. 2024

[23] Text und Wissenschaft (2020) Forschungsmethode Fallstudien (Case Studies) – Text und Wissenschaft. https://www.textundwissenschaft.de/blog/2020/10/16/forschungsmethode-fallstudien-case-studies/. Zugegriffen: 11. Nov. 2024

[24] Lorig K, González V, Laurent D (2012) Manual Kursleitung: Gesund und aktiv leben

[25] Knoll J (1993) Kleingruppenmethoden: Effektive Gruppenarbeit in Kursen, Seminaren, Trainings und Tagungen, 2. Aufl. Beltz, Weinheim

[26] Arnemann P Partnergespräch mit denkanregender Frage. https://dbs-lin.ruhr-uni-bochum.de/lehreladen/lehrformate-methoden/aktivieren-und-motivieren/motiviert-ins-semester/partnergespraech/. Zugegriffen: 12. Nov. 2024

[27] Stumpf S (2019) Quiz – Methodenspicker. https://d-3.germanistik.uni-halle.de/2019/12/quiz-methodenspicker/. Zugegriffen: 12. Nov. 2024

[28] Harder S, Sander B (2019) Angeleitetes Selbststudium

[29] Jiang L, Smith ML, Chen S et al (2015) The role of session zero in successful completion of chronic disease self-management program workshops. Front Public Health. 10.3389/fpubh.2014.00205 25964918 10.3389/fpubh.2014.00205 PMC4410344

[30] Wilsher HS, Brainard J, Loke Y, Salter C (2017) Patient and public involvement in health literacy interventions: a mapping review. Res Involv Engagem 3:31. 10.1186/s40900-017-0081-z 29276627 10.1186/s40900-017-0081-z PMC5738234

[31] Kasberg A, Müller P, Markert C, Bär G (2021) Systematisierung von Methoden partizipativer Forschung. Bundesgesundheitsblatt Gesundheitsforschung Gesundheitsschutz 64:146–155. 10.1007/s00103-020-03267-9 33373015 10.1007/s00103-020-03267-9 PMC7843478

[32] Clar C, Wright MT (2020) Partizipative Forschung im deutschsprachigen Raum – eine Bestandsaufnahme. https://opus4.kobv.de/opus4-ash/frontdoor/index/index/docId/324. Zugegriffen: 17. Sept. 2024

[33] Pratte M‑M, Audette-Chapdelaine S, Auger A‑M, Wilhelmy C, Brodeur M (2023) Researchers’ experiences with patient engagement in health research: a scoping review and thematic synthesis. Res Involv Engagem 9:22. 10.1186/s40900-023-00431-8 37038164 10.1186/s40900-023-00431-8 PMC10088213

[34] Wright MT (2021) Partizipative Gesundheitsforschung: Ursprünge und heutiger Stand. Bundesgesundheitsblatt Gesundheitsforschung Gesundheitsschutz 64:140–145. 10.1007/s00103-020-03264-y 33336312 10.1007/s00103-020-03264-y PMC7843534

